# Supplementary material for: Identification of Human Global, Tissue and Within-Tissue Cell-Specific Stably Expressed Genes at Single-Cell Resolution
Source: Int J Mol Sci. 2022 Sep 6;23(18):10214. doi: 10.3390/ijms231810214 (PMC9499411; doi:10.3390/ijms231810214)
Supplement: Supplementary file 1 [file ijms-23-10214-s001.zip › FigS3.pdf]

A

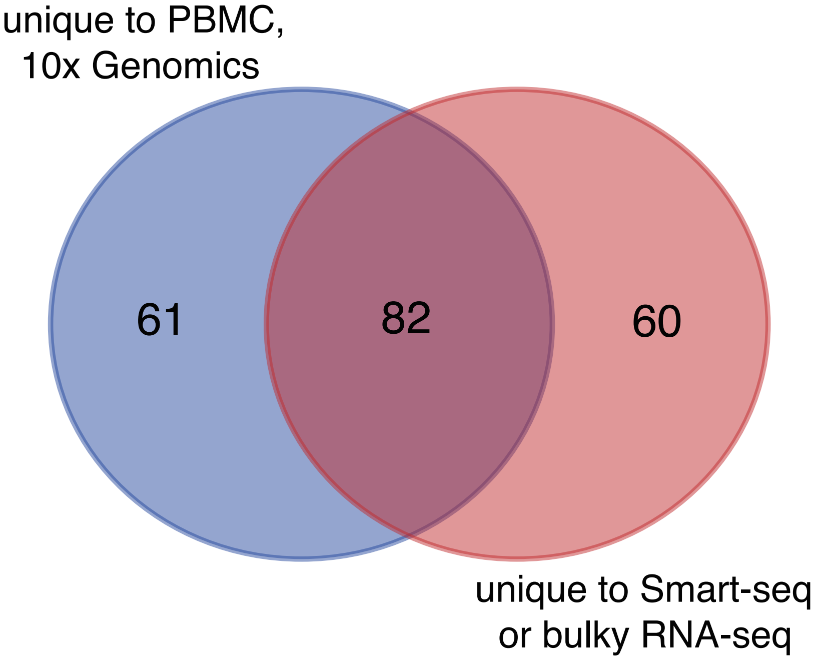
unique to PBMC,
10x Genomics
61
82
60
unique to Smart-seq
or bulky RNA-seq

B

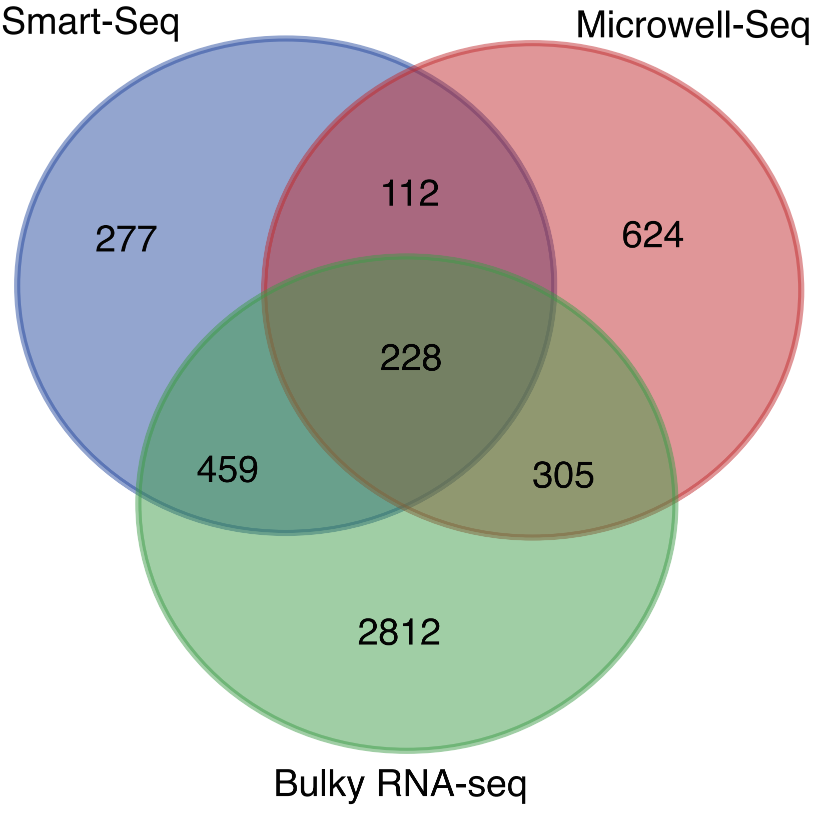
Smart-Seq
Microwell-Seq
277
112
624
228
459
305
2812
Bulky RNA-seq
