# Supplementary material for: Identification of Human Global, Tissue and Within-Tissue Cell-Specific Stably Expressed Genes at Single-Cell Resolution
Source: Int J Mol Sci. 2022 Sep 6;23(18):10214. doi: 10.3390/ijms231810214 (PMC9499411; doi:10.3390/ijms231810214)
Supplement: Supplementary file 1 [file ijms-23-10214-s001.zip › FigS4.pdf]

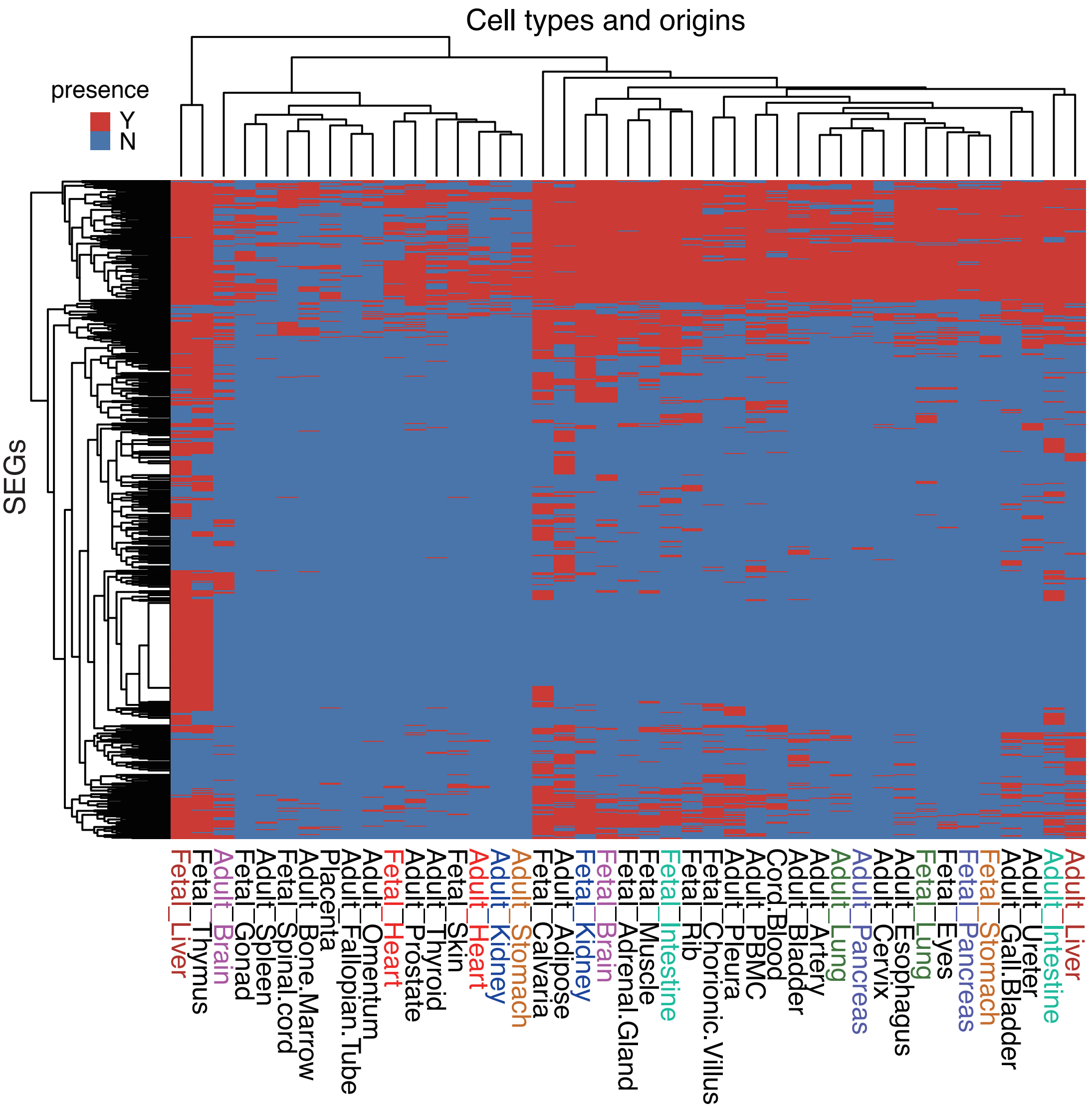
Cell types and origins
presence
Y
N
SEGs
Fetal_Liver
Fetal_Thymus
Adult_Brain
Fetal_Gonad
Adult_Spleen
Fetal_Spinal.cord
Adult_Bone.Marrow
Placenta
Adult_Fallopian.Tube
Adult_Omentum
Fetal_Heart
Adult_Prostate
Adult_Thyroid
Fetal_Skin
Adult_Heart
Adult_Kidney
Adult_Stomach
Fetal_Calvaria
Adult_Adipose
Fetal_Kidney
Fetal_Brain
Fetal_Adrenal.Gland
Fetal_Muscle
Fetal_Intestine
Fetal_Rib
Fetal_Chorionic.Villus
Adult_Pleura
Adult_PBMC
Cord.Blood
Adult_Bladder
Adult_Artery
Adult_Lung
Adult_Pancreas
Adult_Cervix
Adult_Esophagus
Fetal_Lung
Fetal_Eyes
Fetal_Pancreas
Fetal_Stomach
Adult_Gall.Bladder
Adult_Ureter
Adult_Intestine
Adult_Liver
