# Supplementary figures and images for: Identification of Human Global, Tissue and Within-Tissue Cell-Specific Stably Expressed Genes at Single-Cell Resolution
Source: Int J Mol Sci. 2022 Sep 6;23(18):10214. doi: 10.3390/ijms231810214 (PMC9499411; doi:10.3390/ijms231810214)

Number of SEGs

spatio-temporal SEGs  
total number: 408

60  
40  
20  
0

0 10 20 30 40

Number of human tissues

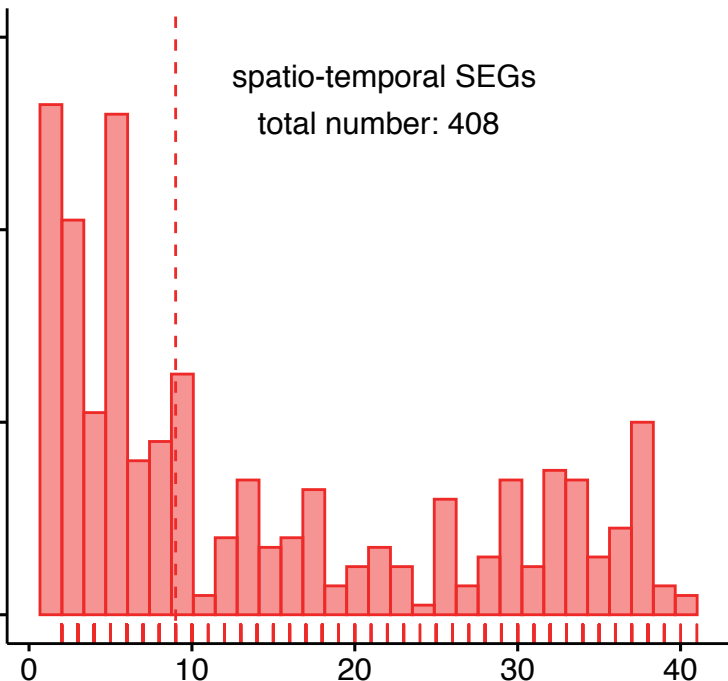

Supplement: Supplementary file 1 [file ijms-23-10214-s001.zip › FigS1.pdf]

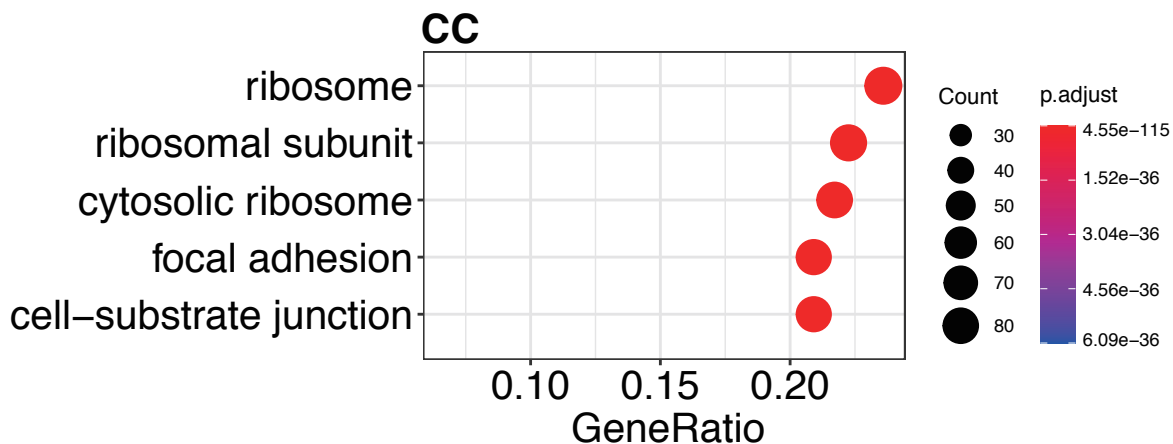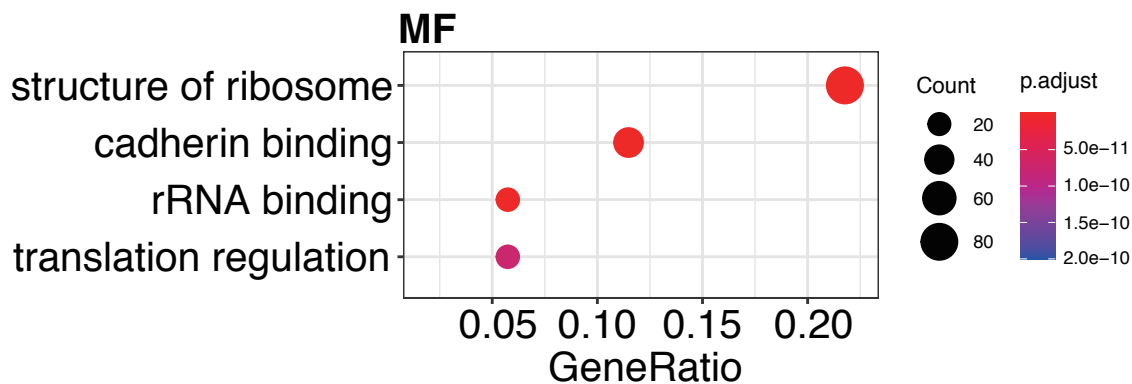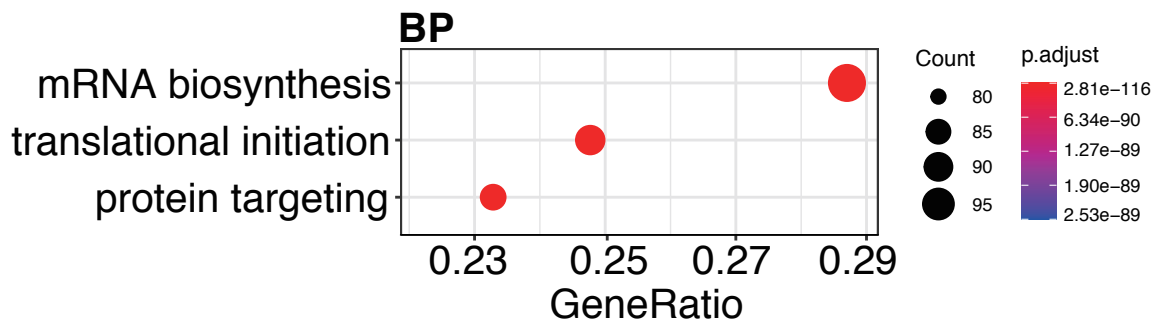

Supplement: Supplementary file 1 [file ijms-23-10214-s001.zip › FigS2.pdf]
